# Eco-friendly biosynthesis of silver nanoparticles using marine-derived *Fusarium exquisite*: optimization, characterization, and evaluation of antimicrobial, antioxidant, and cytotoxic activities

**DOI:** 10.1007/s11274-025-04368-w

**Published:** 2025-05-06

**Authors:** Sally A. Ali, Mohamed E. Osman, Eslam T. Mohamed

**Affiliations:** Department of Botany and Microbiology, Faculty of Science, Helwan University, Cairo, 11795 Egypt

**Keywords:** *Fusarium equiseti*, Silver nanoparticles, Extracellular biosynthesis, Green nanotechnology, Antimicrobial activity, Antioxidant activity, Cytotoxicity, Molecular docking

## Abstract

Fungi serve as efficient biocatalysts for the eco-friendly synthesis of metal nanoparticles, yielding stable and bioactive nanomaterials. In this study, silver nanoparticles were synthesized (AgNPs) using the marine-derived fungus *Fusarium equiseti* and characterized them with various analytical methods. UV–Vis spectroscopy detected a surface plasmon resonance peak at 420 nm, confirming AgNP formation, while X-ray diffraction (XRD) verified their crystalline structure. Scanning electron microscopy (SEM) and transmission electron microscopy (TEM) revealed spherical nanoparticles averaging 50 nm. FTIR analysis confirmed that fungal metabolites cap and stabilize the AgNPs. We optimized extracellular biosynthesis at 30°C, pH 8, and 2 mM AgNO₃ over 72 h. The marine-adapted *F. equiseti* was selected for its robust metabolic capacity and enzyme secretion, enhancing nanoparticle stability and bioactivity. Biological assessments showed that these AgNPs outperformed AgNO₃ in antimicrobial activity, with minimum inhibitory concentrations (MICs) of 6.5 µg/mL against *Staphylococcus aureus* and 7.5 µg/mL against *Escherichia coli*, plus antifungal effects on *Candida albicans* and *Fusarium solani*. They also displayed strong antioxidant activity (IC₅₀ = 56.98 µg/mL) and cytotoxicity against MCF-7 breast cancer cells (IC₅₀ = 24.38 µg/mL). These enhanced effects likely stem from fungal metabolites acting as natural capping agents, minimizing aggregation and boosting bioavailability and biological interactions. Molecular docking studies reinforced these results, revealing strong binding of AgNPs to microbial cell wall proteins, the human apoptotic regulator Bcl-2 (an anticancer target), and the antioxidant enzyme peroxiredoxin-5 (PRDX5). This green synthesis method provides a sustainable, non-toxic alternative to conventional chemical approaches, avoiding hazardous reagents while delivering stable, multifunctional AgNPs. Future in vivo validation and biocompatibility studies are planned to explore the clinical and pharmaceutical potential of *F. equiseti*-derived AgNPs.

**Graphical Abstract:**

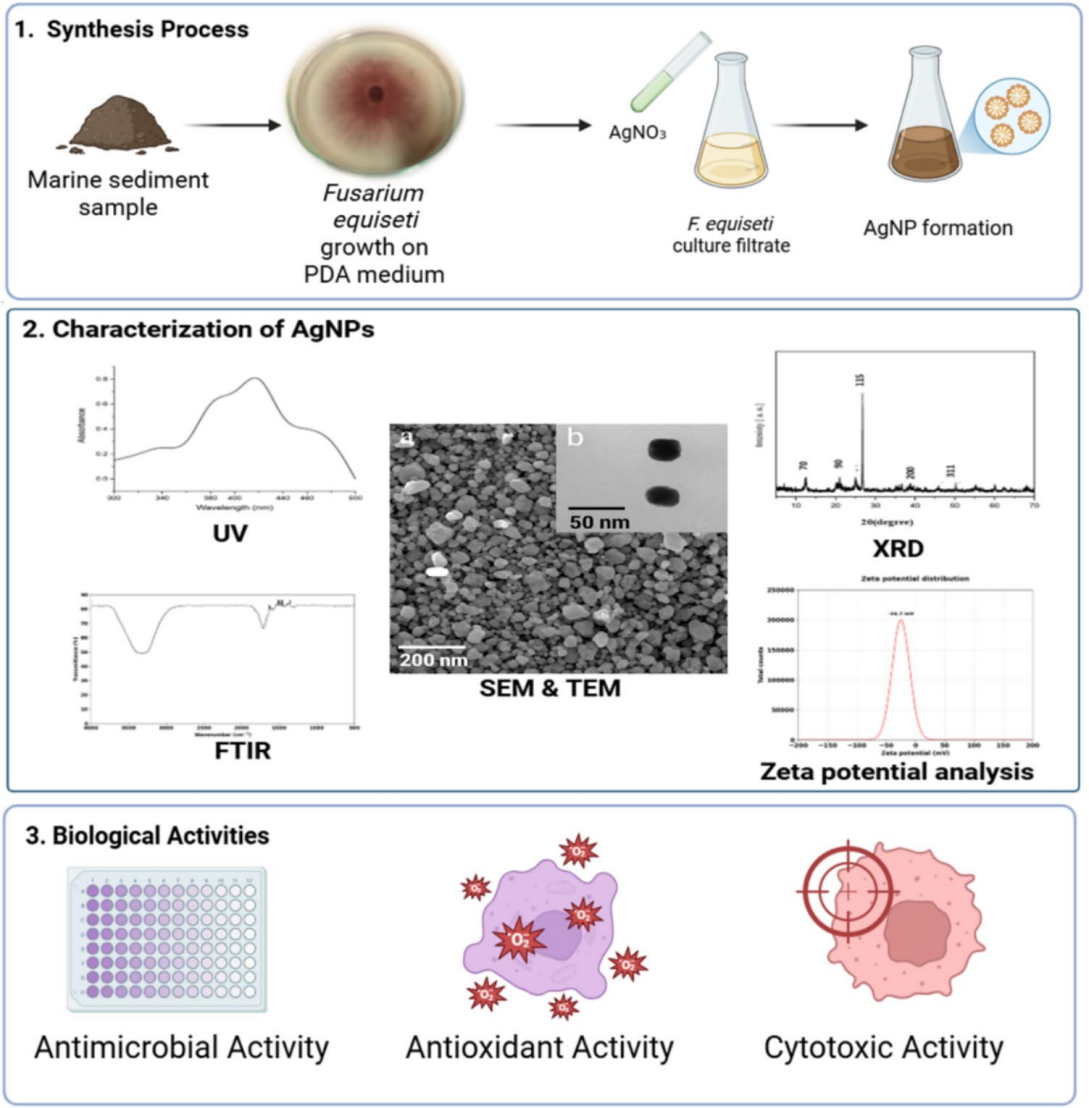

## Introduction

The convergence of nanotechnology and mycology, termed my nanotechnology, is an emerging and promising field that harnesses the vast metabolic potential of fungi for the green synthesis of metal nanoparticles (Sousa et al. 2020; Christopher et al. 2024). Fungi have demonstrated exceptional capability as biological nanofactories due to their rapid growth, ease of cultivation, and ability to secrete a wide array of enzymes and secondary metabolites (Devi et al. 2024). Over recent years, fungal systems have been employed to biosynthesize nanoparticles of various metals—including silver, gold, platinum, and cadmium sulfide (CdS)—with controlled size, shape, and enhanced bioactivity (Guilger-Casagrande and Lima 2019).

The biomedical relevance of fungal-synthesized nanoparticles has become increasingly significant, particularly in addressing two major global health challenges: antimicrobial resistance (AMR) and cancer. AMR is a critical threat to global health, responsible for an estimated 1.27 million deaths annually and undermining the effectiveness of existing antibiotics (WHO 2022). In parallel, cancer remains a leading cause of mortality worldwide, with more than 10 million deaths annually (Sung et al. 2021). These pressing challenges have intensified research into alternative therapeutic strategies, including silver nanoparticles (AgNPs), which are known for their broad-spectrum antimicrobial and anticancer properties. AgNPs can disrupt microbial membranes, generate reactive oxygen species (ROS), inhibit biofilm formation, and induce apoptosis in cancer cells, positioning them as versatile agents in nanomedicine (Alsamhary 2020; Rai et al. 2023).

A synergistic action of extracellular and intracellular biomolecules facilitates fungal-mediated synthesis of AgNPs. Extracellularly, fungi secrete enzymes such as NADH-dependent nitrate reductase, along with proteins, quinones, and polysaccharides, which reduce silver ions (Ag⁺) to elemental silver (Ag⁰) nanoparticles while also stabilizing them (Manjula et al. 2023). These biomolecules act as natural capping agents, preventing nanoparticle aggregation and enhancing their bioavailability. Intracellularly, the fungal cell wall and cytoplasmic enzymes further contribute to nanoparticle formation (Borehalli Mayegowda et al. 2023). Compared to bacteria—which predominantly rely on intracellular pathways and produce fewer extracellular enzymes—fungi provide higher biomass and secrete abundant metabolites, enabling efficient extracellular nanoparticle production with simplified downstream processing (Annamalai et al. 2021). In contrast, plant-mediated synthesis depends largely on phytochemicals like flavonoids and terpenoids, which may yield nanoparticles with broader size distribution and lower stability (Ahmad et al. 2024).

Fungal-based synthesis offers eco-friendly, cost-effective, and scalable alternatives to traditional physical and chemical methods, which typically require high temperatures, pressures, and toxic chemicals (Alsamhary 2020; Kamaruzaman et al. 2022). Moreover, fungal-synthesized AgNPs have demonstrated improved monodispersity, biocompatibility, and bioactivity relative to their chemically synthesized counterparts (Kumari et al. 2023; Mikhailova 2024; Mwangi et al. 2024; Meera et al. 2025). These properties extend the application of fungal nanoparticles beyond medicine to environmental remediation and agriculture (Bhandary et al. 2025; Kirubakaran et al. 2025b).

Among the fungal genera explored, *Fusarium equiseti* remains an underutilized species for nanoparticle biosynthesis. Unlike extensively studied fungi such as *Fusarium oxysporum*, *Aspergillus niger*, and *Penicillium* spp., *F. equiseti* offers untapped potential, especially due to its isolation from marine sediment environments, which promote the production of unique bioactive metabolites under saline stress (Loshchinina et al. 2022; Sudheer et al. 2022). These conditions likely enhance its biosynthetic capacity, enabling the production of stable and biologically potent AgNPs (Roy et al. 2019). The novelty of *F. equiseti* as a marine-derived nanobiofactory, combined with its high enzymatic adaptability, positions it as a promising candidate for sustainable AgNP biosynthesis.

While the antimicrobial and antioxidant properties of silver nanoparticles (AgNPs) are well established, this study offers key advancements by utilizing the marine-derived fungus *F. equiseti* as a novel biocatalyst for nanoparticle biosynthesis. The unique environmental pressures of the marine sediment habitat are hypothesized to stimulate *F. equiseti* to produce distinctive stress-adapted metabolites, which may enhance both the stability and bioactivity of the synthesized AgNPs.

In this work, we report the extracellular biosynthesis of AgNPs using *F. equiseti* isolated from Mediterranean coastal sediments. The biosynthesis process was optimized under defined conditions and systematically correlated with the physicochemical properties and biological activities of the AgNPs. The nanoparticles were comprehensively characterized using X-ray diffraction (XRD), transmission electron microscopy (TEM), scanning electron microscopy (SEM), UV–Vis spectroscopy, and Fourier-transform infrared spectroscopy (FTIR). The biosynthesized AgNPs were subsequently evaluated for their antimicrobial, antioxidant, and cytotoxic activities, confirming their multifunctional bioactivity. To further elucidate the mechanism of action, molecular docking simulations were performed to assess the interactions between AgNPs and key microbial cell wall-associated enzymes, as well as human apoptotic regulatory proteins, providing mechanistic insights that are rarely integrated into fungal-mediated nanoparticle research. Collectively, this study positions *F. equiseti* as a promising marine-derived nanobiofactory for the sustainable synthesis of bioactive AgNPs with potential applications in addressing antimicrobial resistance and cancer.

## Materials and methods

### Isolation and purification of Fusarium equiseti from a marine sediment sample

A sediment sample was collected from the Mediterranean coast of northern Egypt for fungal isolation using the serial dilution technique on potato dextrose agar (PDA) plates. To enhance fungal recovery, we suspended 1 g of wet sediment in 9 mL of sterilized distilled water and incubated it at 60 °C for 40 min to minimize bacterial contamination. A 100 µL aliquot was spread onto PDA plates containing potato extract (200 g/L), glucose (10 g/L), and agar (16 g/L), supplemented with streptomycin (50 µg/mL) and penicillin (100 µg/mL) to suppress bacterial growth. Serial dilutions was prepared (10⁻^1^, 10⁻^2^, 10⁻^3^) with sterilized seawater to ensure fungal isolation, incubated the plates at 25 °C in the dark, and monitored fungal growth for seven days. Individual colonies were subcultured onto fresh PDA plates for purification, transferring actively growing hyphal tips to isolate a single strain. The purified isolate was stored at 4 °C on PDA slants for subsequent studies (Farouk et al. 2024).

### Morphological and molecular characterization of Fusarium equiseti

The identification of the isolated fungal strain was performed using a combination of morphological and molecular approaches. Initial morphological characterization was carried out using the mycological keys of Arifah et al. (2023), along with cultural and conidial characteristics. The micromorphological features of the fungal isolate were examined using slide culture techniques and observed under a light microscope (Optika Microscope, Italy). The isolate was maintained on Sabouraud dextrose agar (SDA) at 28 °C and stored at 4 °C for further studies.

For molecular identification, Internal Transcribed Spacer (ITS) region sequencing was employed to confirm the morphological classification of *Fusarium equiseti*. Genomic DNA was extracted using the Patho-gene-spin DNA/RNA extraction kit (Intron Biotechnology, Korea) following the manufacturer’s protocol. The fungal strain was cultivated on Czapek's yeast extract agar (CYA) for *Penicillium* species and V8 juice agar for *Alternaria* species, with incubation at 28 °C for seven days (Al Mousa et al. 2021). The extracted DNA was submitted to SolGent Company (Daejeon, South Korea) for PCR amplification and sequencing of the ITS region.The amplification reaction was performed using universal fungal primers ITS1 (5'-TCC GTA GGT GAA CCT GCG G-3') and ITS4 (5'-TCC TCC GCT TAT TGA TAT GC-3'). Following amplification, Sanger sequencing was performed using the same primers. The obtained sequences were analyzed using the Basic Local Alignment Search Tool (BLAST) from the National Center for Biotechnology Information (NCBI) database to determine sequence similarity. Phylogenetic analysis was conducted using MegAlign (DNA Star) software version 5.05 to assess the evolutionary relationships of *Fusarium equiseti* with closely related species (Cheruiyot et al. 2024).

### Extracellular biosynthesis of silver nanoparticles using Fusarium equiseti

The extracellular biosynthesis of silver nanoparticles (AgNPs) was conducted utilizing the culture filtrate of *F. equiseti*. The term "extracellular" denotes that nanoparticle formation occurred in the fungal culture supernatant, external to the fungal biomass, facilitated by the secreted bioactive metabolites and enzymes. Specifically, *F. equiseti* is known to release extracellular reductase enzymes (e.g., NADH-dependent nitrate reductase), proteins, phenolic compounds, and polysaccharides into the surrounding medium, which collectively serve as reducing and stabilizing agents. For biosynthesis, *F. equiseti* was cultured in Sabouraud dextrose broth (SDB) at 28 ± 2 °C under shaking conditions (150 rpm) for 5 days to promote maximum secretion of extracellular metabolites. The culture broth was subsequently filtered using Whatman No. 1 filter paper to obtain a sterile cell-free supernatant. To this filtrate, an aqueous solution of silver nitrate (AgNO₃) was added to a final concentration of 1 mM and incubated in the dark at 28 ± 2 °C under static conditions. The biosynthetic reaction was monitored periodically, with the visual transition from pale yellow to dark brown confirming the extracellular reduction of Ag⁺ ions to elemental silver nanoparticles (Ag⁰) (Hulikere and Joshi 2019).

To ensure the validity of the biosynthesis process and exclude abiotic factors, a negative control was included, consisting of 1 mM AgNO₃ added to uninoculated, autoclaved SDB medium under identical incubation conditions. No color change or nanoparticle formation was observed in this control. All synthesis experiments were performed under aseptic conditions, with autoclaved media and sterile equipment, to prevent external contamination. The extracellular approach was selected for its advantages over intracellular synthesis, including ease of nanoparticle recovery, reduced processing steps, and the inherent functionalization of AgNPs by fungal biomolecules, enhancing their colloidal stability and biological activities (Rai et al. 2021).

### Structural and morphological characterization of silver nanoparticles

The formation of silver nanoparticles (AgNPs) was initially confirmed by a visible color change in the reaction mixture, where the fungal filtrate and AgNO₃ solution transitioned from pale yellow to brown, indicating the bioreduction of Ag⁺ ions into elemental silver (Ag⁰) nanoparticles. The synthesis of AgNPs was further verified using ultraviolet–visible (UV–Vis) spectroscopy with a Shimadzu UV-2600 spectrophotometer (Shimadzu Corporation, Japan), which detected a distinct surface plasmon resonance (SPR) peak within the 300–500 nm wavelength range, characteristic of AgNPs. The synthesized AgNPs were purified by centrifugation at 15,000 × g for 15 min using an Eppendorf 5804R centrifuge (Eppendorf, Germany) and repeatedly washed with deionized water to eliminate residual ions and impurities. The purified nanoparticles were then subjected to comprehensive physicochemical characterization. The crystallinity and phase composition of the AgNPs were analyzed using X-ray diffraction (XRD) with a Rigaku RINT2000 vertical goniometer (Rigaku Corporation, Japan), operated at 70 kV and 200 mA with Cu Kα radiation (λ = 1.5405 Å), scanning over a 2θ range of 10°–70°. Morphological and surface characteristics were evaluated by scanning electron microscopy (SEM) and transmission electron microscopy (TEM). For SEM, the AgNP suspension was drop-cast onto carbon-coated copper grids, air-dried, and examined using a Quanta FEG 250 SEM (Thermo Fisher Scientific, USA) to assess particle size distribution and surface morphology. High-resolution imaging was performed via transmission electron microscopy (TEM) using a HITACHI H-800 TEM (Hitachi, Japan) at 200 kV to confirm the shape and structural integrity of the synthesized nanoparticles. Additionally, Fourier-transform infrared (FTIR) spectroscopy (PerkinElmer Spectrum Two, USA) was employed to identify functional groups involved in nanoparticle stabilization and surface functionalization. Zeta potential analysis was carried out using a Malvern Zetasizer Nano ZS (Malvern Instruments, UK) to determine the colloidal stability of the AgNPs in aqueous suspension (Fath-Alla et al. 2024).

### Optimization of silver nanoparticle biosynthesis parameters

One-variable-at-a-time (OVAT) approach was used to assess the influence of temperature, pH, and silver nitrate (AgNO₃) concentration on nanoparticle formation. The effect of temperature was evaluated by incubating the reaction mixture at 15, 20, 25, 30, 35, 40, and 45°C while maintaining constant pH and AgNO₃ concentration. Similarly, the impact of pH on AgNP biosynthesis was assessed by adjusting the reaction medium to pH 3, 4, 5, 6, 7, 8, and 9 using 0.1 M HCl or 0.1 M NaOH. The influence of silver ion concentration was examined by testing AgNO₃ at different concentrations (1, 1.5, 2, 2.5, 3, and 3.5 mM). The formation and stability of AgNPs under different conditions were monitored by measuring surface plasmon resonance (SPR) at 420 nm using a Shimadzu UV-2600 spectrophotometer (Shimadzu Corporation, Japan) (Salem et al. 2024).

### Antimicrobial activity of silver nanoparticles

The antimicrobial activity of the biosynthesized silver nanoparticles (AgNPs) from *Fusarium equiseti* was evaluated using a broth microdilution assay to determine the minimum inhibitory concentration (MIC) against bacterial and fungal pathogens, including *Fusarium solani*, *Bacillus subtilis*, *Pseudomonas aeruginosa*, *Staphylococcus aureus*, *Escherichia coli*, and *Candida albicans*. Bacterial strains (*P. aeruginosa* ATCC 25619, *B. subtilis* ATCC 6633, *S. aureus* LC189114, and *E. coli* OK087362) were cultured overnight in nutrient broth at 37 °C. The bacterial suspensions were adjusted to an optical density (OD) of 0.2 (~ 2 × 10^4^ CFU/mL). Spore suspensions of *F. solani* and *C. albicans* were also adjusted to 2 × 10^4^ CFU/mL. AgNPs were serially diluted to final concentrations ranging from 0.125 to 64 μg/mL in Czapek's Yeast Broth (CYB) for fungi and in nutrient broth for bacteria. In sterile 96-well microtiter plates, 100 µL of each AgNP dilution was mixed with 100 µL of microbial suspension. Plates were incubated at 37 °C for bacteria and 28 °C for fungi. Streptomycin (for bacteria) and fluconazole (for fungi) were used as positive controls, while silver nitrate (AgNO₃) was included as a comparative control. After 24 h (bacteria) or 72 h (fungi), microbial growth was assessed, and MIC values were recorded as the lowest AgNP concentration that visibly inhibited microbial growth (Mwangi et al. 2024).

### Evaluation of the antioxidant activity of silver nanoparticles

The antioxidant activity of biosynthesized silver nanoparticles (AgNPs) was assessed using the 2,2-diphenyl-1-picrylhydrazyl (DPPH) radical scavenging assay. A 0.5 mL solution of 0.15 mM DPPH was prepared and mixed with AgNPs dissolved in methanol. Ascorbic acid was used as a positive control. The reaction mixture was incubated at room temperature for 30 min in the dark to allow the reduction of the DPPH radical by AgNPs. After incubation, absorbance was measured at 517 nm using a UV–Vis spectrophotometer (Shimadzu UV-2600, Shimadzu Corporation, Japan). The percentage of DPPH radical scavenging activity was calculated using the following equation: Reduction of the DPPH radical (%) = [(Control absorbance—Control sample absorbance)/ (Control absorbance)] × 100. Higher scavenging activity indicated a stronger antioxidant potential of the AgNPs (Bhavi et al. 2024).

### Evaluation of the cytotoxic effects of silver nanoparticles on MCF-7 Cells

The cytotoxic activity of biosynthesized silver nanoparticles (AgNPs) against MCF-7 human breast cancer cells was assessed using the MTT (3-(4,5-dimethylthiazol-2-yl)-2,5-diphenyltetrazolium bromide) assay. MCF-7 cells were seeded at a density of 1 × 10^4^ cells per well in a 96-well microtiter plate and allowed to adhere overnight. The cells were then exposed to varying concentrations of AgNPs (0, 10, 20, 30, 40, 50, and 60 μg/mL) and incubated for 24 h at 37°C in a humidified atmosphere containing 5% CO₂. Following incubation, 100 µL of MTT solution (0.5 mg/mL in phosphate-buffered saline, PBS, pH 7.2) was added to each well, and the plate was further incubated for 3 h at 37 °C to allow the formation of insoluble purple formazan crystals. The MTT solution was carefully removed, and 50 µL of dimethyl sulfoxide (DMSO) was added to dissolve the formazan. Absorbance was measured at 570 nm using a microplate reader (Bio-Rad iMark™, USA). Cell viability was expressed as a percentage relative to the untreated control group, and cytotoxicity was determined based on the decrease in viability in response to increasing AgNP concentrations (Al-Ziyadi et al. 2024).

### Molecular docking study of silver nanoparticles against microbial and human target proteins

A spherical silver nanoparticle (AgNP) model, with a diameter of 50 nm, was constructed using Materials Studio 2020 based on the face-centered cubic (FCC) crystal structure of bulk silver. To ensure the structural and energetic stability of the model, energy minimization was performed using the density functional theory (DFT) approach. The nanoparticle surface was subsequently functionalized with citrate ions to improve solubility, biocompatibility, and colloidal stability by preventing nanoparticle aggregation in aqueous environments. The stability of the citrate-functionalized AgNPs was further verified through molecular dynamics simulations under simulated physiological conditions. Molecular docking simulations were performed using Molegro Virtual Docker (MVD, version 6.0) to evaluate the interactions between the citrate-functionalized AgNP model and selected microbial and human proteins. Protein structures were retrieved from UniProt and the Protein Data Bank (PDB) and were prepared by removing water molecules, adding polar hydrogens, and assigning partial atomic charges using standard MVD protocols. The active sites of each protein were automatically predicted and grid boxes were generated to focus docking runs on biologically relevant regions (Nayel et al. 2024).

The protein targets were chosen based on their essential roles in microbial survival, cell wall biosynthesis, and human cellular processes. For bacterial strains, targets involved in peptidoglycan biosynthesis and remodeling were selected, including the cell wall-associated protease (P54423) in *B. subtilis*, peptidoglycan D,D-transpeptidase PbpC (Q9I1K1) in *P. aeruginosa*, glycyl-glycine endopeptidase LytM (O33599) in *S. aureus*, and peptidoglycan D,D-transpeptidase MrdA (P0AD65) in *E. coli*. These enzymes are critical to bacterial cell wall integrity, making them attractive targets for antimicrobial action (Hugonneau-Beaufet et al. 2023; Piatek et al. 2023; Darrouzet et al. 2024; Zhao et al. 2025). For fungal pathogens, 2-methylcitrate synthase, mitochondrial (C7C435) in *F. solani,* and the cell wall integrity transcriptional regulator CAS5 (Q5AMH6) in *C. albicans* were selected due to their roles in fungal metabolism and structural maintenance (Xiong et al. 2021).

In addition to microbial targets, human proteins were included to evaluate the potential antioxidant and apoptotic activities of AgNPs. For the antioxidant mechanism, pyridoxine 5'-phosphate synthase (PdxS, P0A794), a key enzyme in oxidative stress resistance pathways, was selected to assess how AgNPs might disrupt cellular redox homeostasis (Rivero et al. 2024). For the cytotoxicity mechanism, the anti-apoptotic protein Bcl-2 (P10415) was selected due to its involvement in regulating mitochondrial-mediated apoptosis in cancer cells (Harikrishnan et al. 2021). The inclusion of these targets was intended to provide mechanistic insights into the dual antimicrobial and anticancer effects of the biosynthesized AgNPs.

### Statistical analysis

All experiments were performed in triplicate, and the data are presented as mean ± standard deviation (SD). Statistical significance was assessed using one-way analysis of variance (ANOVA) followed by Tukey’s post hoc test for multiple comparisons. A p-value of < 0.05 was considered statistically significant. The IC₅₀ values for DPPH radical scavenging activity and MCF-7 cytotoxicity assays were calculated using nonlinear regression analysis fitted to a sigmoidal dose–response model. All statistical analyses were performed using GraphPad Prism (version 9.0) and SPSS software (version 22.0).

## Results

### Fungal isolation, identification, and biosynthesis of silver nanoparticles by Fusarium equiseti

**Fig. 1:**
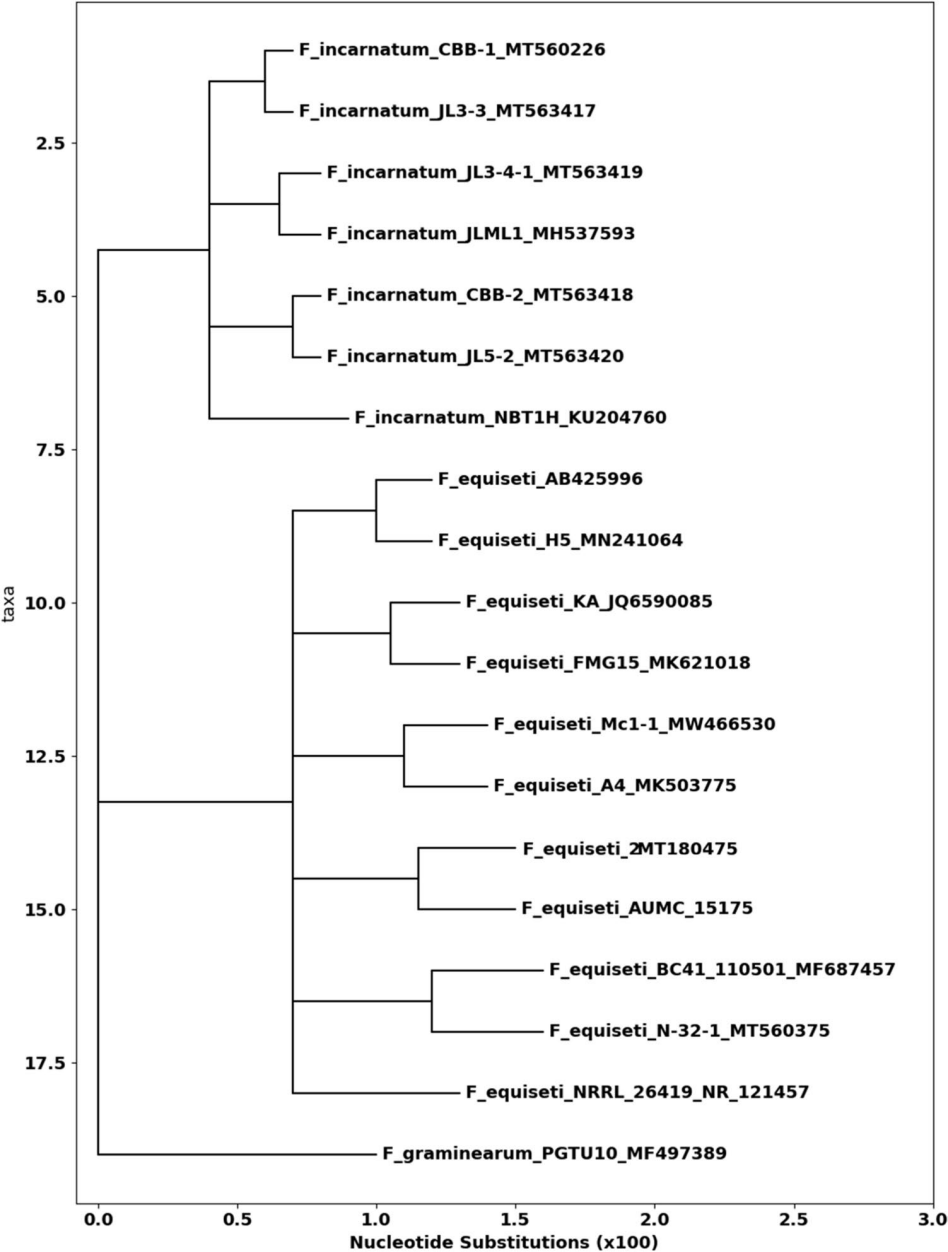
Phylogenetic analysis of *F. equiseti* showing its alignment with closely related *Fusarium* strains from GenBank. The isolate AUMC 15175 exhibited 100% sequence identity with multiple *F. equiseti* strains

Sediment samples from the Mediterranean coast of northern Egypt yielded several fungal isolates: *Scopulariopsis brevicaulis*, *Macrophomina phaseolina*, *Fusarium semitectum*, *Mucor hiemalis*, *Curvularia spicifera*, *F. equiseti*, and *Chaetomium* sp. Only *F. equiseti* demonstrated extracellular biosynthesis of silver nanoparticles (AgNPs). Morphological analysis identified *F. equiseti* by its white peripheral hyphae, rapid colony growth, and velvety, floccose aerial mycelium with pale-to-rose pigmentation, typical of the genus. Molecular characterization via ITS region sequencing confirmed this, with BLAST analysis showing 100% similarity to *F. equiseti* strains (GenBank: AUMC 15175). Phylogenetic analysis closely clustered our isolate with other *F. equiseti* strains (Fig. 1).

### Biosynthesis and physicochemical characterization of silver nanoparticles

**Fig. 2:**
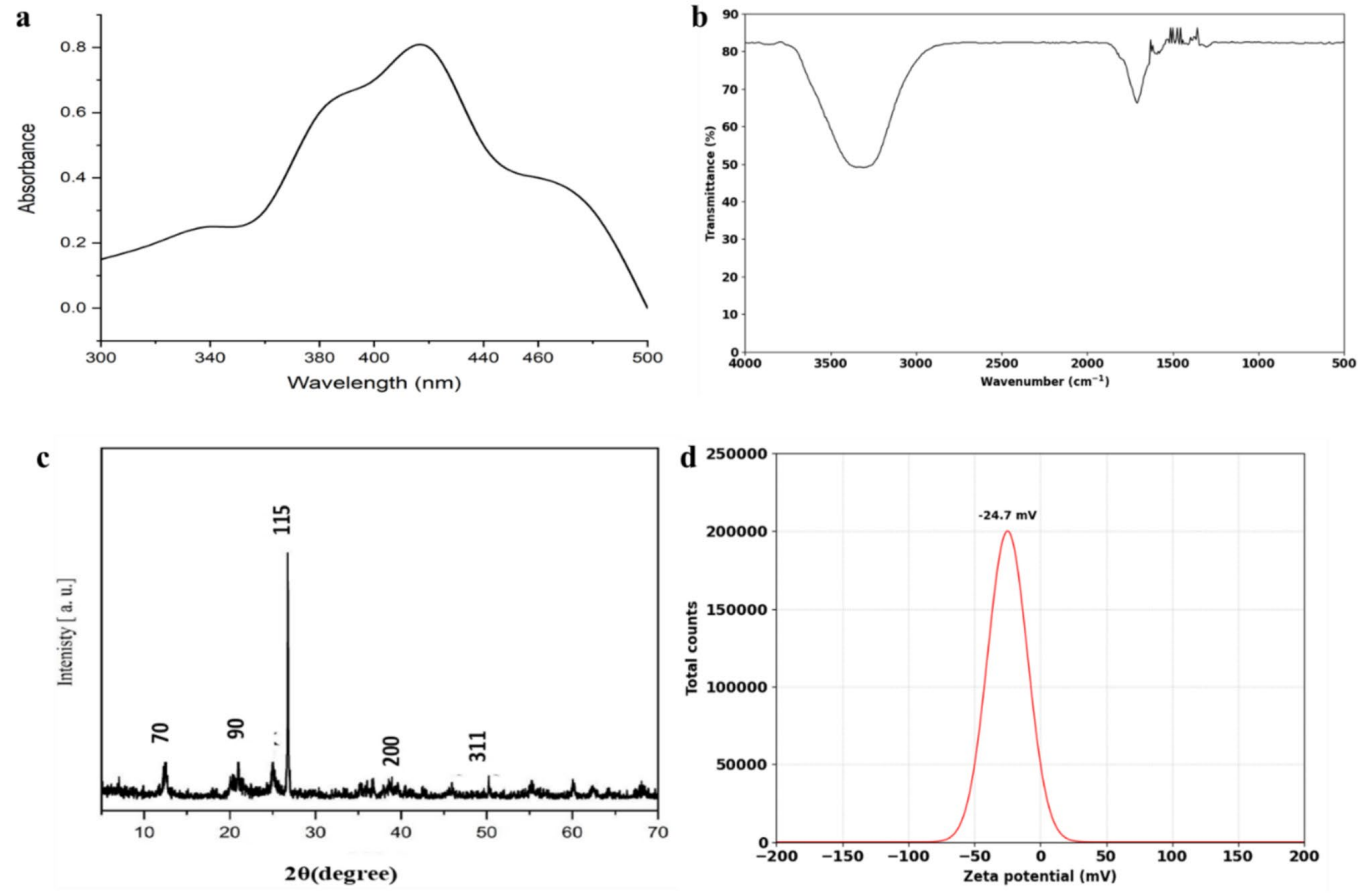
Characterization of biosynthesized silver nanoparticles (AgNPs) produced by *F. equiseti*: **a** UV–Vis absorption spectrum of AgNPs showing surface plasmon resonance (SPR) at ~ 420 nm, **b** FTIR spectrum indicating functional groups responsible for nanoparticle stabilization, **c** XRD pattern confirming the crystalline structure of AgNPs, **d** Zeta potential analysis showing colloidal stability with a zeta potential of –24.7 mV

**Fig. 3:**
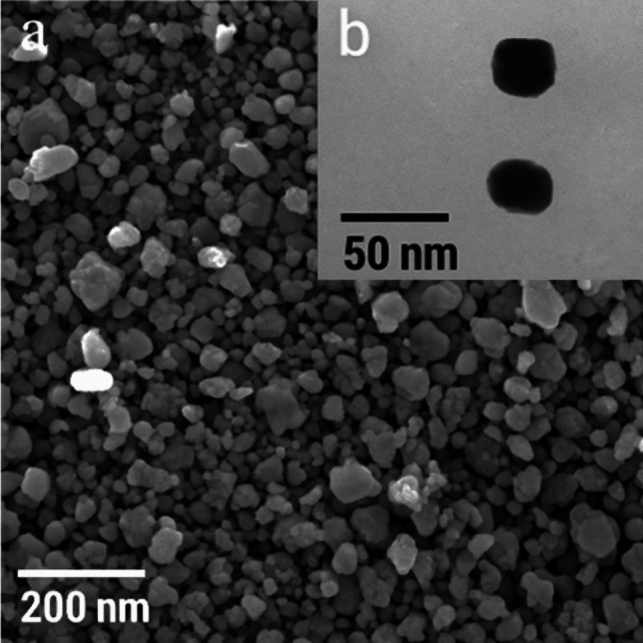
SEM and TEM images: **a** SEM image of silver nanoparticles synthesized using *F. equiseti* filtrate (magnification: 2000 ×); **b** TEM image showing spherical morphology of AgNPs

Extracellular AgNP biosynthesis was evident from a color shift from pale yellow to dark brown after adding 1 mM AgNO₃ to *F. equiseti* culture filtrate, reflecting surface plasmon resonance (SPR) typical of AgNP formation. UV–Vis spectroscopy confirmed this with a sharp peak at 420 nm (Fig. 2a), indicating spherical AgNPs and stable colloidal dispersion. FTIR analysis identified key functional groups involved in reduction and stabilization: bands at 3295 cm⁻^1^ (O–H stretching, hydroxyls), 1636 cm⁻^1^ (amide I, proteins), and 1393 cm⁻^1^ (C-O stretching, polysaccharides) (Fig. 2b), pointing to fungal proteins, polysaccharides, and phenolics as capping agents. XRD revealed peaks at (111), (200), (220), and (311) planes, confirming a face-centered cubic (FCC) crystalline structure (Fig. 2c). Zeta potential measured –24.7 mV (Fig. 2d), suggesting good colloidal stability via electrostatic repulsion and fungal biomolecule stabilization. SEM and TEM showed predominantly spherical AgNPs averaging 50 nm, with uniform distribution and minimal aggregation (Fig. 3).

### Optimization of silver nanoparticle biosynthesis parameters

**Fig. 4:**
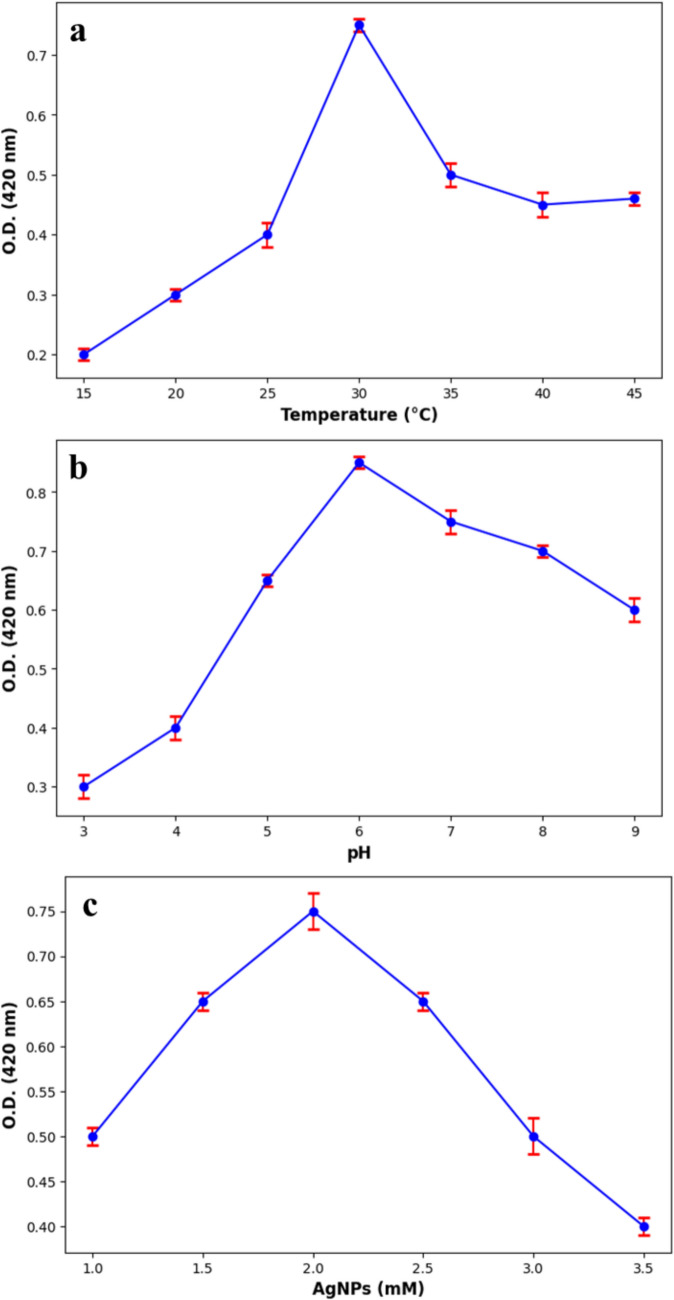
Optimization of biosynthesis of AgNPs by *F*. *equiseti*. **a** Temperature, **b** pH, **c** Silver nitrate concentration

Biosynthesis parameters were optimized using a one-variable-at-a-time (OVAT) approach. The maximum AgNP yield and stability were achieved at 30°C, pH 6, and 2 mM AgNO₃. Deviations from these conditions resulted in decreased nanoparticle formation or increased aggregation (Fig. 4a–c).

#### Antimicrobial activity of silver nanoparticles

**Fig. 5:**
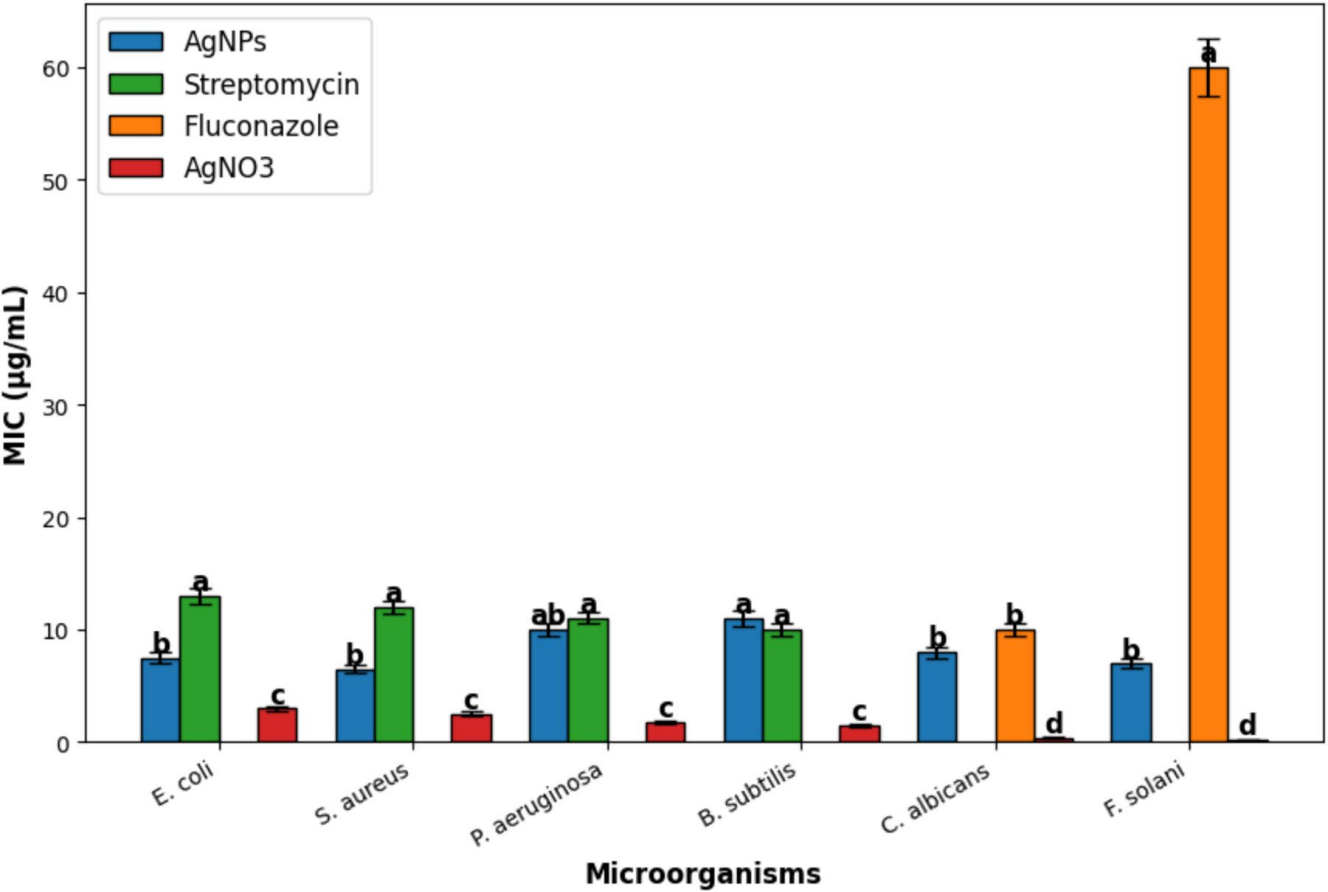
Minimum inhibitory concentrations (MICs) of AgNPs, Streptomycin, Fluconazole, and AgNO₃ against various microorganisms (values represent mean ± standard deviation, different letters indicate significant differences at p < 0.05)

The antimicrobial activity of AgNPs was further evaluated by determining MIC values against various microbial strains, and the results were compared with standard antibiotics (Streptomycin and Fluconazole) as well as silver nitrate (AgNO₃). The results indicate that AgNPs exhibit strong antimicrobial activity against both bacterial and fungal strains. Notably, *S. aureus* and *E. coli* were highly susceptible to AgNPs, with MIC values of 6.5 and 7.5 µg/mL, respectively. The antifungal activity against *C. albicans* (MIC = 8 µg/mL) was comparable to Fluconazole (MIC = 10 µg/mL. Similarly, *F. solani* exhibited high sensitivity to AgNPs (MIC = 7 µg/mL) (Fig. 5).

#### Evaluation of the antioxidant activity of silver nanoparticles

**Fig. 6:**
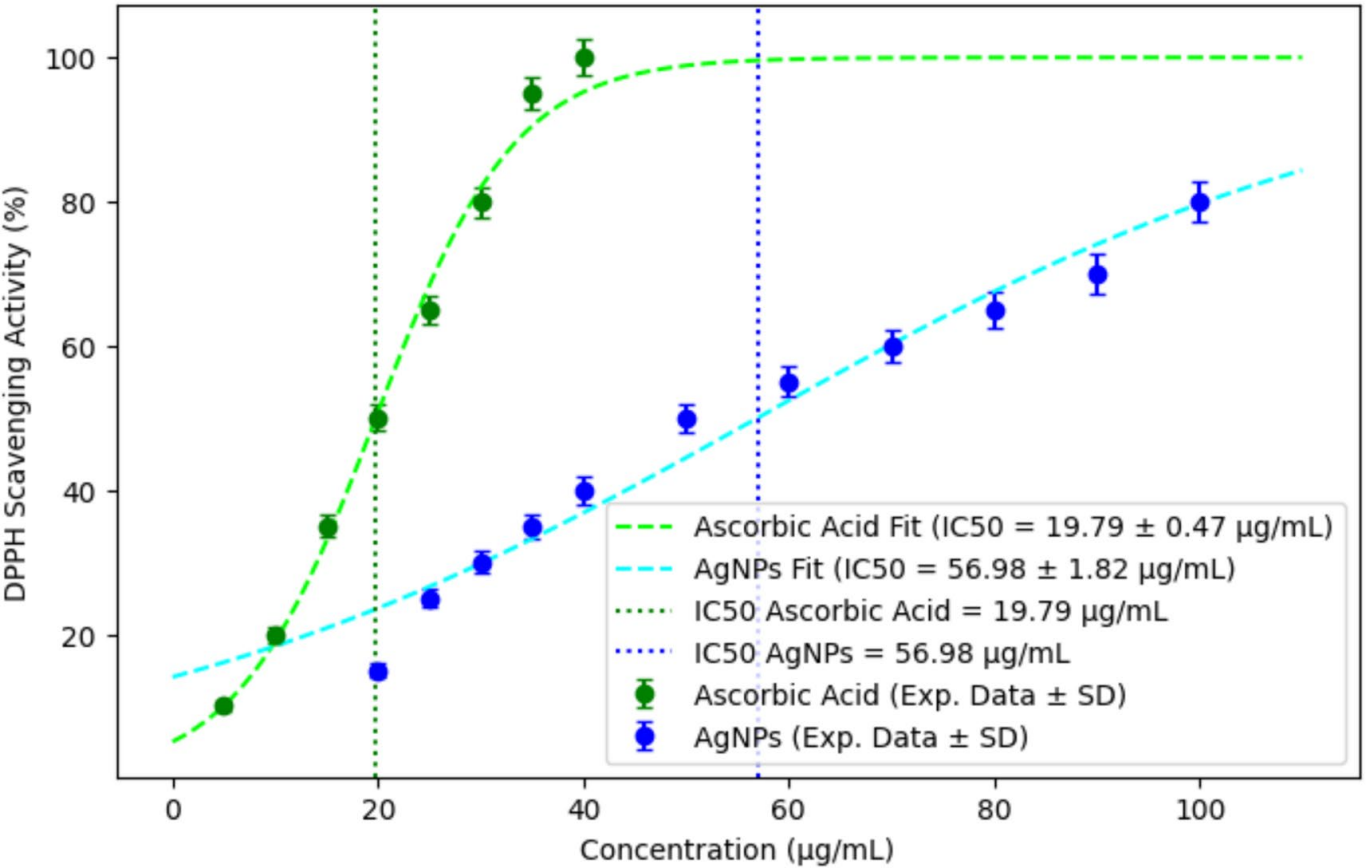
Dose-dependent DPPH radical scavenging activity of biosynthesized AgNPs.

The DPPH (2,2-diphenyl-1-picrylhydrazyl) radical scavenging assay was employed to evaluate the antioxidant potential of biosynthesized AgNPs and the aqueous extract of *F. equiseti*. The results demonstrated that AgNPs exhibited significant antioxidant activity, with radical scavenging ability increasing in a dose-dependent manner. The IC₅₀ value for ascorbic acid was determined to be 19.79 µg/mL, while the IC₅₀ value for AgNPs was 56.98 µg/mL (Fig. 6).

### Evaluation of the cytotoxic effects of silver nanoparticles on MCF-7 Cells

**Fig. 7:**
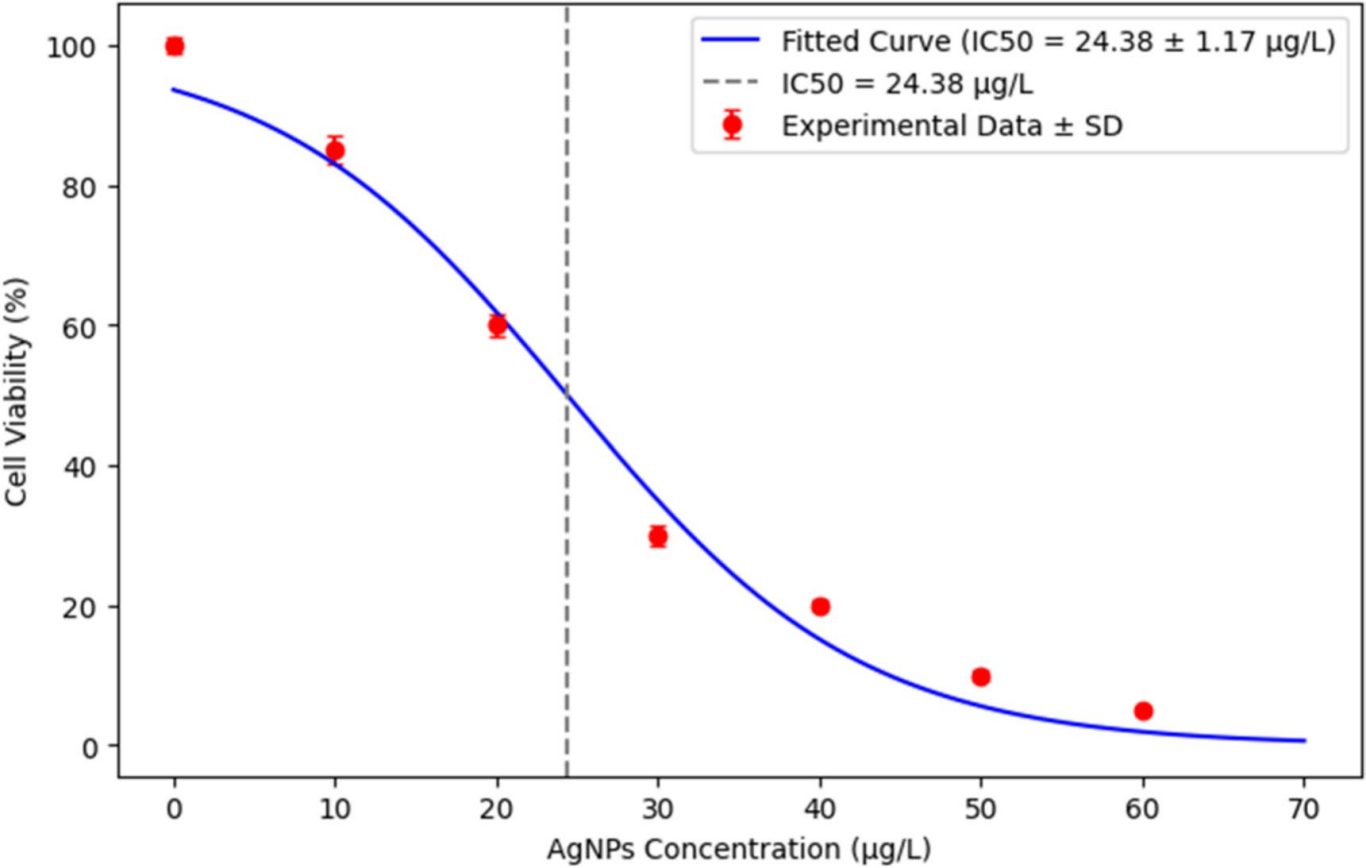
Dose–response curve for AgNPs cytotoxicity

The results demonstrated a concentration-dependent cytotoxic effect of AgNPs against MCF-7 breast cancer cells, as illustrated in Fig. 7. The half-maximal inhibitory concentration (IC₅₀), which represents the concentration of AgNPs required to inhibit 50% of cell viability, was calculated as 24.38 μg/mL.

### Molecular docking study of silver nanoparticles against microbial and human target proteins

**Fig. 8:**
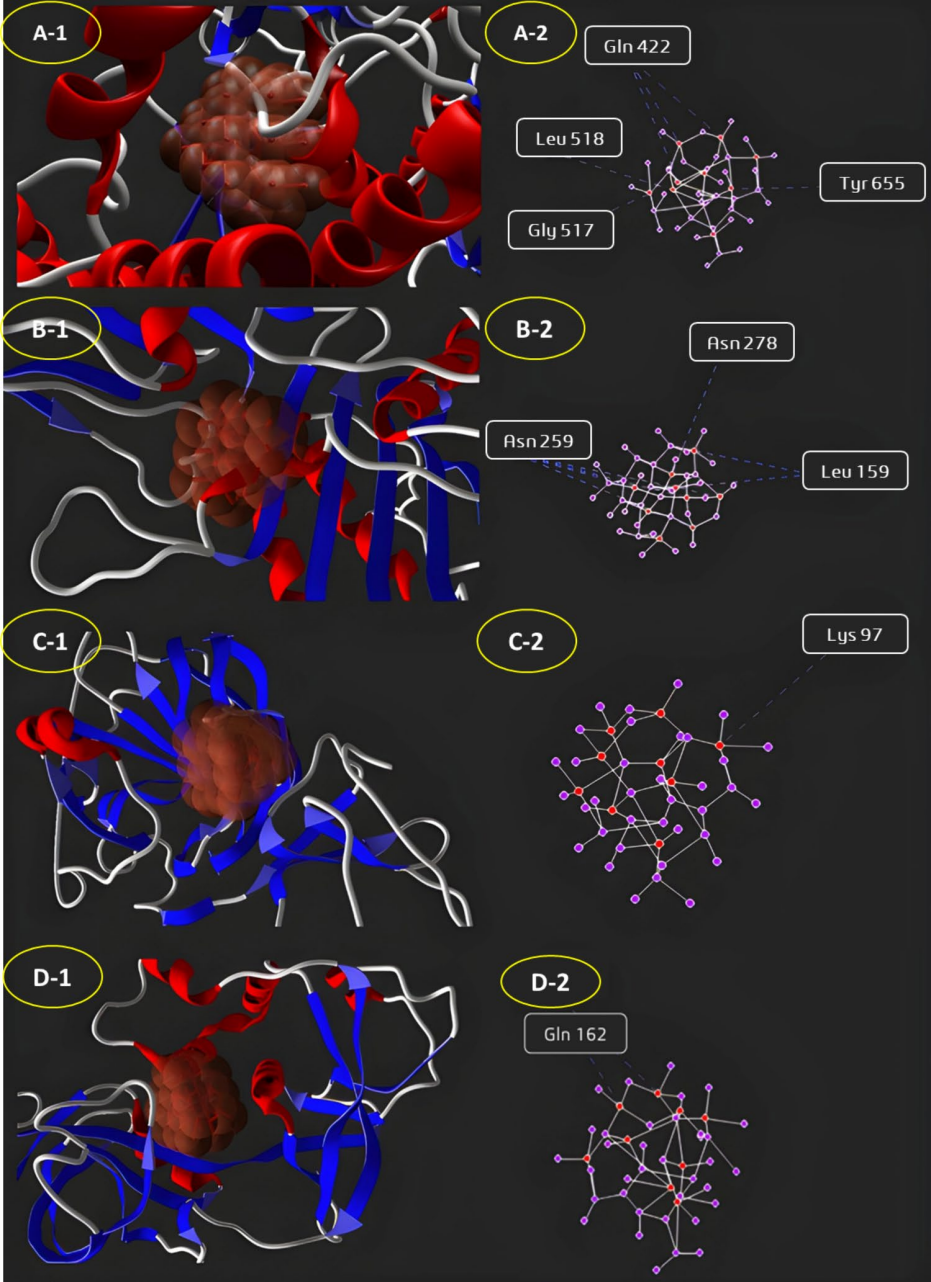
Molecular docking interactions of silver nanoparticles with microbial and human target proteins. The figure represents docking studies of AgNPs with **A** *B. subtilis*—CWP (Cell Wall Protease), **B** *P. aeruginosa*—PbpC (Peptidoglycan Transpeptidase), **C** *S. aureus*—LytM (Glycyl-glycine Endopeptidase), **D** *E. coli*—MrdA (Peptidoglycan Transpeptidase)

**Fig. 9:**
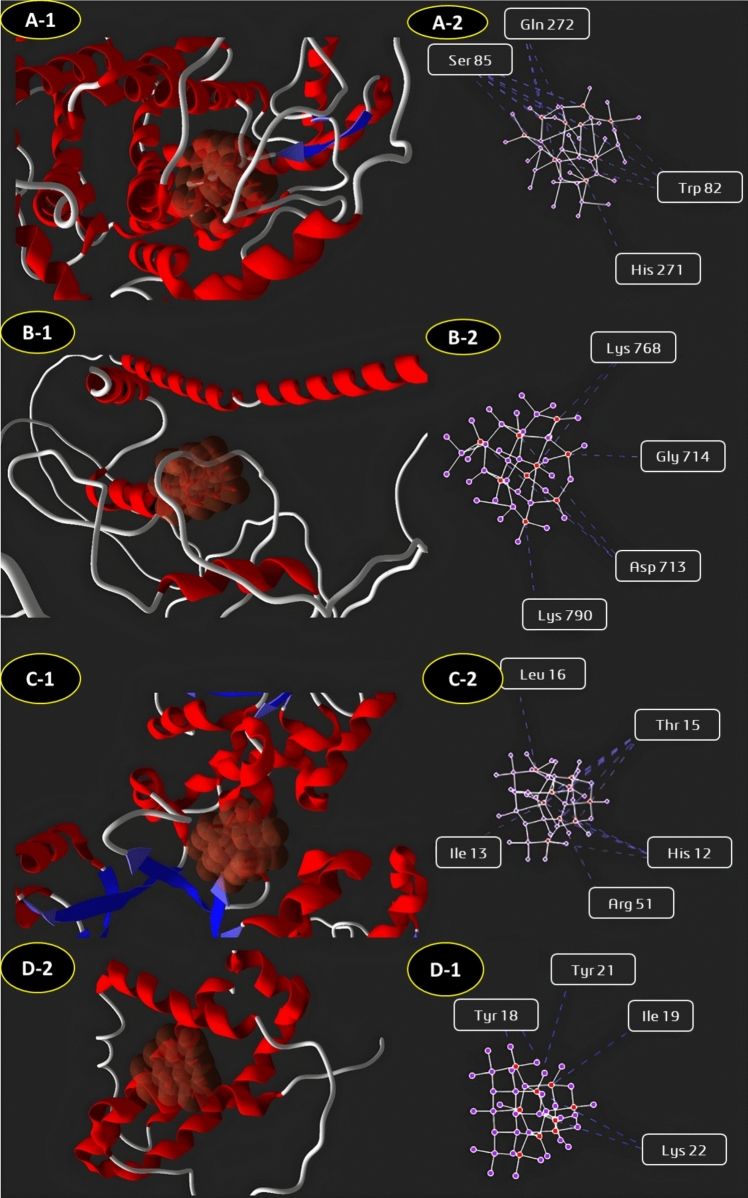
Molecular docking interactions of silver nanoparticles with microbial and human target proteins. The figure represents docking studies of AgNPs with **A** *F. solani*—MCS (2-Methylcitrate Synthase),(**B** *C. albicans*—CAS5 (Cell Wall Integrity Regulator), **C** antioxidant Target—PdxS (Pyridoxine Synthase), **D** apoptosis Target—Bcl-2 (Apoptosis Regulator)

The highest binding affinity was observed with *C. albicans* CAS5 (−62.45 kcal/mol), a key regulator of fungal cell wall integrity. Structural analysis revealed hydrogen bonding and hydrophobic interactions with crucial active site residues. AgNPs also showed significant binding with *B. subtilis* protease (−60.26 kcal/mol) and *P. aeruginosa* PbpC (−56.59 kcal/mol), proteins essential for bacterial survival and cell wall synthesis. Electrostatic and hydrophobic interactions in these binding pockets indicate strong inhibitory potential. For cytotoxic targets, AgNPs exhibited strong binding with Bcl-2 (−58.21 kcal/mol), an anti-apoptotic protein (Figs. 8 and 9).

## Discussion

Fungal-mediated biosynthesis of silver nanoparticles (AgNPs), particularly using *Fusarium* species, has gained recognition as a sustainable and efficient nanotechnology strategy, capitalizing on fungi’s robust metabolic capabilities (Loshchinina et al. 2022). This study introduces the novel use of marine-derived *Fusarium equiseti* for extracellular AgNP synthesis, followed by comprehensive characterization and evaluation of their antimicrobial, antioxidant, and cytotoxic properties. While prior research has established fungi as effective nanobiofactories due to their high metabolic activity and secretion of bioactive secondary metabolites (Salem and Fouda 2021; Nassar et al. 2023; Herrera Pérez et al. 2024), our work stands out as the first to harness *F. equiseti* from Mediterranean coastal sediments. This marine adaptation likely enriches the fungal filtrate with unique stress-induced metabolites, enhancing AgNP stability and bioactivity. Recent reviews underscore the growing prominence of green synthesis in producing multifunctional nanomaterials for biomedical applications (Kirubakaran et al. 2025a), and our findings expand this scope, offering a sustainable alternative with significant potential to address antimicrobial resistance and cancer.

The successful extracellular biosynthesis of AgNPs underscores *F. equiseti*’s efficacy in reducing silver ions through secreted biomolecules, such as enzymes and polysaccharides. A visible color shift from pale yellow to dark brown, coupled with a surface plasmon resonance (SPR) peak at 420 nm, confirmed AgNP formation—results consistent with those reported for other *Fusarium* species (Sivalingam and Pandian 2024; Verma et al. 2024). X-ray diffraction (XRD) analysis further validated the nanoparticles’ face-centered cubic (FCC) crystalline structure, affirming their purity and stability, in line with *Cladosporium oxysporum*-mediated AgNP synthesis (Isaq et al. 2023). Compared to chemical methods, which often yield less stable nanoparticles due to the absence of biogenic stabilizers (Tortella et al. 2020), our fungal approach offers a sustainable route to high-quality AgNPs, a strategy increasingly validated by green nanotechnology advancements (Mikhailova 2024). The AgNPs biosynthesized by *F. equiseti* in this study displayed a uniform spherical morphology and an average size of ~ 50 nm, as verified by SEM and TEM analyses. This size slightly exceeds the 10–40 nm range reported for AgNPs from *Aspergillus brasiliensis* (Moreno-Vargas et al. 2023), potentially reflecting the marine strain’s unique metabolic profile. Remarkably, these nanoparticles exhibited strong colloidal stability, with a zeta potential of –24.7 mV—comparable to AgNPs from *Exserohilum rostrata* (Bagur et al. 2020), surpassing the stability of chemically synthesized AgNPs, which often aggregate without biogenic stabilizers due to reliance on toxic agents like sodium borohydride or hydrazine (Tortella et al. 2020). The fungal-mediated approach here leverages *F. equiseti*’s natural proteins, enzymes, and polysaccharides as capping agents, preventing agglomeration while enhancing biocompatibility and bioactivity (Sudheer et al. 2022). Such properties align with recent insights into sustainable nanomaterials for biomedical and environmental applications (Kirubakaran et al. 2025a), reinforcing the advantages of green synthesis.

Optimizing biosynthesis parameters is critical to maximize the yield, stability, and bioactivity of silver nanoparticles. Here, *F. equiseti* achieved optimal AgNP production at 30°C, pH 8, and 2 mM AgNO₃ over 72 h, yielding stable, spherical nanoparticles with potent bioactivity. These conditions align with prior fungal-mediated syntheses, such as those using *Fusarium oxysporum* and *Aspergillus fumigatus*, which thrive at slightly alkaline pH (7–9) and moderate temperatures (28–30 °C) (Tariq et al. 2022). Unlike *Penicillium* spp., which require shorter incubation periods (48–60 h), the 72-h duration for *F. equiseti* may reflect its marine adaptation, potentially slowing metabolic rates under saline stress (Bihal et al. 2023). Temperature optimization at 30 °C proved ideal—below 25 °C, reaction rates dropped, while above 40 °C, protein denaturation triggered aggregation (Tariq et al. 2022). Similarly, pH 8 balanced enzyme activity and stability, as acidic conditions (pH < 5) inhibited reduction, and overly alkaline pH (> 8) induced clustering (Kumari et al. 2023). The Booking2 mM AgNO₃ concentration struck an effective equilibrium: lower levels (< 1 mM) failed to drive complete reduction, while higher levels (> 3 mM) caused uncontrolled nucleation and aggregation (Manjula et al. 2023). This balance, supported by physiological conditions, produced uniform, bioactive AgNPs (Qin et al. 2020), a finding echoed in recent studies on microbial nanoparticle synthesis. These optimizations highlight *F. equiseti*’s potential as a scalable, eco-friendly nanobiofactory.

Silver nanoparticles (AgNPs) are well-known for their antimicrobial prowess, primarily through disrupting cell membranes, inhibiting enzymes, and interfering with DNA replication (Rosli et al. 2021). In this study, *F. equiseti*-derived AgNPs demonstrated robust antibacterial and antifungal activity, with standout efficacy against *Candida albicans* (MIC = 8 µg/mL) and *Bacillus subtilis* (MIC not specified but significant). This broad-spectrum potency mirrors findings from recent research, affirming AgNPs’ effectiveness against Gram-positive and Gram-negative bacteria as well as fungi (Mikhailova 2024). The antimicrobial mechanism hinges on AgNPs’ capacity to generate reactive oxygen species (ROS), inducing oxidative stress that damages microbial cell components. By interacting with bacterial membranes, AgNPs increase permeability, trigger intracellular leakage, and ultimately cause lysis (Boateng and Catanzano 2020). Compared to AgNO₃ alone, our AgNPs exhibited superior MICs (e.g., 6.5 µg/mL vs. higher values for AgNO₃), likely due to fungal capping enhancing bioavailability—a key advantage over chemical alternatives, as supported by green synthesis studies using plant fluids (Sabira et al. 2025).

A pivotal antimicrobial mechanism of AgNPs involves their affinity for sulfur- and phosphorus-containing biomolecules in microbial cells. Silver ions bind avidly to thiol (-SH) groups in essential enzymes and proteins, disrupting metabolic processes and causing microbial death (More et al. 2023). Additionally, AgNPs impair DNA replication by targeting nucleic acid phosphate groups, halting cell division and proliferation (Mikhailova 2024). Microbial susceptibility varies by structure: Gram-negative bacteria like *Pseudomonas aeruginosa* resist AgNPs due to an outer membrane barrier, whereas Gram-positive species, such as *Bacillus subtilis* and *S. aureus*, with thicker peptidoglycan layers, are more vulnerable, as evidenced by our stronger inhibition of these strains (Khaldoun et al. 2024). Against *Candida albicans*, our AgNPs exhibited potent antifungal activity (MIC = 8 µg/mL), likely by destabilizing chitin- and glucan-rich cell walls and targeting ergosterol, a critical membrane component, leading to permeability and cell death (Do et al. 2025). These findings highlight *F. equiseti*-derived AgNPs as versatile antimicrobial agents, surpassing traditional antibiotics in potency against resistant pathogens, a potential further explored in recent green synthesis research (Sabira et al. 2025).

Silver nanoparticles’ antioxidant properties are increasingly valued for their potential to combat oxidative stress-related disorders (Balkrishna et al. 2021). Here, *F. equiseti*-derived AgNPs displayed significant, dose-dependent free radical scavenging, with an IC₅₀ of 56.98 µg/mL—evidence of their ability to neutralize reactive oxygen species (ROS) linked to cellular damage and aging (Flieger et al. 2021). This activity stems from AgNPs donating electrons to stabilize free radicals, mitigating oxidative stress (Bedlovičová et al. 2020). Compared to ascorbic acid (IC₅₀ = 19.79 µg/mL), our AgNPs are less potent but align with other biologically synthesized nanoparticles, such as those from *Syzygium caryophyllatum* (Bhavi et al. 2024) and *Nepenthes mirabilis* pitcher fluid (Sabira et al. 2025), suggesting fungal capping enhances antioxidant capacity. This positions *F. equiseti* AgNPs as promising candidates for oxidative stress-related therapeutic applications, consistent with sustainable nanomaterial developments (Rajkumar et al. 2025).

The cytotoxic potential of AgNPs is increasingly recognized for its promise in cancer therapy (Miranda et al. 2022). Here, *F. equiseti*-derived AgNPs exerted potent, dose-dependent cytotoxicity against MCF-7 breast cancer cells, with an IC₅₀ of 24.38 µg/mL—comparable to fungal AgNPs tested on MCF-7, HepG2, and A549 lines (Singh et al. 2021). This effect arises from AgNPs generating reactive oxygen species (ROS), inducing oxidative stress, mitochondrial dysfunction, and apoptosis. Excessive ROS disrupts homeostasis, causing lipid peroxidation, DNA damage, and protein oxidation, culminating in programmed cell death (Sanati et al. 2022)—a widely documented mechanism in AgNP-mediated cancer cell toxicity. Additionally, AgNPs impair mitochondrial electron transport, depleting ATP and triggering an energy crisis that amplifies apoptosis (Guo et al. 2023), while also arresting cell cycle progression to curb proliferation (Mobaraki et al. 2022). Relative to chemically synthesized AgNPs, our fungal-derived nanoparticles may benefit from marine metabolites enhancing bioavailability, a hypothesis supported by recent plant-mediated synthesis studies (Sabira et al. 2025). Though these in vitro results highlight *F. equiseti* AgNPs’ multifunctional bioactivity, in vivo studies and cytotoxicity assays on normal cells are essential to validate therapeutic efficacy and safety, addressing scalability and biocompatibility challenges (Kirubakaran et al. 2025a).

Molecular docking elucidated the mechanisms underlying *F. equiseti*-derived AgNPs’ bioactivity against microbial and human targets (Othman and Kamel 2025). The nanoparticles showed strong binding to microbial proteins, notably *C. albicans* CAS5 (−62.45 kcal/mol), a key regulator of fungal cell wall integrity, suggesting AgNPs disrupt synthesis and repair, heightening susceptibility to osmotic stress and damage (Álvarez-Chimal et al. 2022). Similarly, a high affinity for *Bacillus subtilis* cell wall-associated protease (−60.26 kcal/mol) implies interference with protein turnover critical for survival. In human targets, AgNPs bound robustly to Bcl-2 (−58.21 kcal/mol), an anti-apoptotic protein, indicating potential to shift the apoptotic balance and induce cancer cell death, consistent with reports of mitochondrial disruption (Guo et al. 2023; Takáč et al. 2023). Interaction with pyridoxine 5'-phosphate synthase (P0A794) suggests AgNPs amplify oxidative stress in microbes, bolstering antimicrobial effects (Sabira et al. 2025). These docking insights—unique in integrating marine fungal AgNPs with such targets—substantiate the dual antimicrobial and anticancer potential, aligning with recent molecular docking studies on green-synthesized AgNPs (Sabira et al. 2025), and position *F. equiseti* AgNPs as a multifunctional nanomaterial.

## Conclusion

This study marks a significant advancement in green nanotechnology by demonstrating the eco-friendly, extracellular biosynthesis of silver nanoparticles (AgNPs) using the marine-derived fungus *Fusarium equiseti*, a previously underutilized species. The resulting AgNPs, characterized as stable, spherical particles (~ 50 nm) with a zeta potential of –24.7 mV, exhibited exceptional bioactivity: antimicrobial MICs as low as 6.5 µg/mL against *Staphylococcus aureus* and 7.5 µg/mL against *Escherichia coli*, antifungal effects against *C. albicans* (MIC = 8 µg/mL), antioxidant activity (IC₅₀ = 56.98 µg/mL), and cytotoxicity against MCF-7 breast cancer cells (IC₅₀ = 24.38 µg/mL). Molecular docking further elucidated their mechanisms, revealing strong binding affinities (e.g., –62.45 kcal/mol with *C. albicans* CAS5 and –58.21 kcal/mol with human Bcl-2), underscoring their dual antimicrobial and anticancer potential. The marine origin of *F. equiseti* likely enhances these properties through unique stress-adapted metabolites, distinguishing this approach from conventional fungal syntheses and chemical methods. While these in vitro results are promising, they remain preliminary, necessitating in vivo validation, biocompatibility assessments (e.g., toxicity on normal cells, hemocompatibility), and ROS quantification to confirm oxidative stress mechanisms. This work not only expands the repertoire of fungal nanobiofactories but also offers a sustainable, scalable platform for developing multifunctional nanomaterials, with potential to address antimicrobial resistance and cancer therapy in clinical and industrial contexts.

## Data Availability

The datasets generated and analyzed during the current study are available from the corresponding author on reasonable request.
